# Interaction of circadian clock proteins PER2 and CRY with BMAL1 and CLOCK

**DOI:** 10.1186/1471-2199-9-41

**Published:** 2008-04-22

**Authors:** Sonja Langmesser, Tiziano Tallone, Alain Bordon, Sandro Rusconi, Urs Albrecht

**Affiliations:** 1 Department of Medicine, Division of Biochemistry, University of Fribourg, Rue du Musée 5, 1700 Fribourg, Switzerland; 2 PolyGene AG, Riedmattstrasse 9, 8153 Rümlang, Switzerland; 3 Friedrich Miescher Institute for Biomedical Research, Novartis Research Foundation, Maulbeerstrasse 66, CH-4058 Basel, Switzerland; 4 Dipartimento dell’educazione, della cultura e dello sport, Divisione della cultura e degli studi universitari, Viale S. Franscini 30a, 6501 Bellinzona, Switzerland

## Abstract

**Background:**

Circadian oscillation of clock-controlled gene expression is mainly regulated at the transcriptional level. Heterodimers of CLOCK and BMAL1 act as activators of target gene transcription; however, interactions of PER and CRY proteins with the heterodimer abolish its transcriptional activation capacity. PER and CRY are therefore referred to as negative regulators of the circadian clock. To further elucidate the mechanism how positive and negative components of the clock interplay, we characterized the interactions of PER2, CRY1 and CRY2 with BMAL1 and CLOCK using a mammalian two-hybrid system and co-immunoprecipitation assays.

**Results:**

Both PER2 and the CRY proteins were found to interact with BMAL1 whereas only PER2 interacts with CLOCK. CRY proteins seem to have a higher affinity to BMAL1 than PER2. Moreover, we provide evidence that PER2, CRY1 and CRY2 bind to different domains in the BMAL1 protein.

**Conclusion:**

The regulators of clock-controlled transcription PER2, CRY1 and CRY2 differ in their capacity to interact with each single component of the BMAL1-CLOCK heterodimer and, in the case of BMAL1, also in their interaction sites. Our data supports the hypothesis that CRY proteins, especially CRY1, are stronger repressors than PER proteins.

## Background

Circadian rhythms are recurring fluctuations with a period of about 24 hours that can be observed in the physiology and behavior of most living organisms from cyanobacteria to humans [1]. They are controlled by an autonomous circadian clock, which can be synchronized to the environmental day-night cycle. In mammals, the suprachiasmatic nucleus (SCN), a structure in the ventral part of the hypothalamus, appears to be the main coordinator of the circadian timing system [2,3] synchronizing peripheral clocks present in all tissues throughout the body [4].

The oscillatory mechanism underlying the circadian clock has been unraveled by means of genetic analysis in *Drosophila *and mammals [5]. In the latter, a heterodimeric complex of two transcriptional activators, CLOCK and BMAL1, binds to E-box enhancer elements present in the promoters of target genes and thereby activates the expression of three *Period *(*Per1*, *Per2 *and *Per3*) and two *Cryptochrome *genes (*Cry1 *and *Cry2*). PER and CRY proteins translocate to the nucleus where CRY proteins act as potent (and PER proteins as mild) inhibitors of CLOCK-BMAL1-induced transcription [6,7]. The positive (CLOCK-BMAL1) and negative (CRY, PER) arms of the feedback loop are coupled via the nuclear orphan receptor REV-ERBα [8] generating a stabilized feedback loop that drives recurrent rhythms in mRNA and protein levels of clock components.

Transcriptional reporter assays, yeast two-hybrid screens and co-immunoprecipitation experiments have been successfully used to identify molecular interactions of clock components at the protein level [6,7,9-12]. Interactions of BMAL1 with CLOCK, NPAS2, DEC1 and DEC2 have been identified. Furthermore it has been suggested that the transactivation activity of BMAL1 is mediated by interaction with CREB binding protein (CBP) or p300 [13].

Many different approaches have been employed to characterize the interactions between the repressors PER2, CRY1 and CRY2 and the BMAL1-CLOCK heterodimer, but still the picture is far from being clear. It is thought that CRY1 plays a key role in repressing the transcriptional activation potential of the heterodimer, and recently, various attempts have been made to elucidate the mechanism by which interaction of CRY1 with BMAL1 and/or CLOCK inhibits transcription [14-17]. However, many of these studies use multimeric protein complexes, which do not always satisfactorily identify the exact interactions between two individual components of the complex.

We decided to choose a complementary approach and to investigate the interactions of PER2, CRY1 and CRY2 with BMAL1 and CLOCK using a mammalian two-hybrid system where we only overexpressed two of the components – one part of the activating heterodimer and one repressor – at a time. All interactions identified in the two-hybrid system were confirmed by co-immunoprecipitation. Our results indicate that in our conditions, CRY1, CRY2 and PER2 proteins interact with BMAL1 by binding to different sites of BMAL1, but that only PER2 interacts with CLOCK alone. Moreover, in keeping with the idea that the CRY proteins are more potent inhibitors than PER2, we found that CRY1 and CRY2 both modify the interaction between PER2 and BMAL1, but not vice versa.

## Results and Discussion

### Interaction of PER2, CRY1 and CRY2 with BMAL1

**Figure 1:**
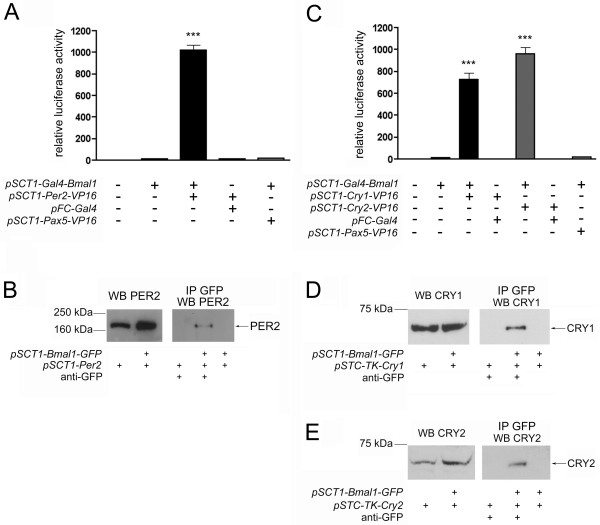
**PER2, CRY1 and CRY2 all interact with BMAL1**. (A) and (C) HER911 cells were co-transfected with 3 μg *pFR-luc*, 0.1 μg *pCMV-lacZ *and the indicated expresssion plasmids. For each experiment (n = 6), values obtained for cells transfected with the luciferase reporter alone were set to 1. *** p < 0.001 as determined by student's t-test compared to all other columns. (B), (D) and (E) HER911 cells were co-transfected with *pSCT1-Bmal1-GFP *and *pSCT1-Per2 *(B), *pSTC-TK-Cry1 *(D) or *pSTC-TK-Cry2 *(E). Total cell extracts (left panels) or immunoprecipitates using an anti-GFP antibody (right panels) were subjected to Western blotting using antibodies against PER2, CRY1 and CRY2, respectively. Blots are representative results from one experiment, all co-immunoprecipitations were repeated twice. WB Western Blot, IP immunoprecipitation.

We first sought to identify the interactions between each of the repressors with BMAL1 using a mammalian two-hybrid system. HER911 cells were co-transfected with *Bmal1 *fused to the Gal4 DNA binding domain (Gal4 DBD) and either *Per2*, *Cry1 *or *Cry2 *fused to the activation domain of the viral protein VP16. In this system, an interaction of the two clock components reconstitutes a functional transcription factor that will activate a luciferase reporter under the control of a GAL4-based promoter. For GAL4-BMAL1/PER2-VP16, a 1000-fold increase in luciferase activity was observed (Fig. 1A). Similar values were obtained for GAL4-BMAL1/CRY1-VP16 and GAL4-BMAL1/CRY2-VP16 (750- and 950-fold, respectively; Fig. 1C). However, only a minimal activation of the reporter was seen when GAL4-BMAL1 was expressed alone or together with an unrelated fusion protein (PAX5-VP16) or when the VP16 fusions were co-transfected with the Gal4 DBD alone (Fig. 1A, C).

Both PER2 (Fig. 1B) and the CRY proteins (Fig. 1D, E) could be co-immunoprecipitated from extracts of HER911 cells co-transfected with *Bmal1-GFP *and the respective interaction partner using an anti-GFP antibody. We therefore conclude that all three proteins are able to bind to BMAL1, which is in line with previous reports [6,15,18,19]. The fact that each of the three repressors can interact with BMAL1 and thus has the potential to influence BMAL1-CLOCK mediated transcription would also explain why *Per2*, *Cry1 *and *Cry2 *mutant mice all display an altered expression of genes regulated by BMAL1 and CLOCK [18,20-22].

### Interactions with CLOCK

**Figure 2:**
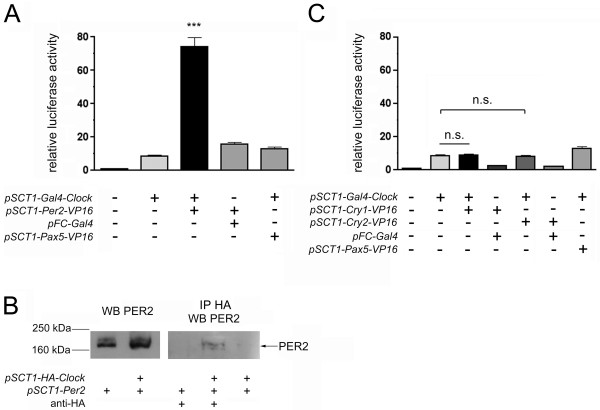
**Only PER2, but neither CRY1 nor CRY2, interacts with CLOCK**. (A) and (C) HER911 cells were co-transfected with *pFR-luc*, *pCMV-lacZ *and the indicated expresssion plasmids. For each experiment (n = 6), values obtained for cells transfected with the luciferase reporter alone were set to 1. *** p < 0.001 as determined by student's t-test compared to all other columns; n.s. not significant. (B) HER911 cells were co-transfected with *pSCT1-HA-Clock *and *pSCT1-Per2*. Total cell extracts (left panel) or immunoprecipitates using an anti-HA antibody (right panel) were subjected to Western blotting using an antibody against PER2. Blots are representative results from one experiment, the co-immunoprecipitation was repeated twice. WB Western Blot, IP immunoprecipitation.

When repeating the same experiments using *Gal4-Clock *instead of *Gal4-Bmal1*, we still found a 75-fold transactivation of the reporter in the case of co-transfection with *Per2 *(Fig. 2A). However, co-transfection with *Cry1/2-VP16 *did not induce luciferase more than transfection with *Gal4-Clock *alone (Fig. 2C). Using an anti-HA antibody, were we able to co-immunoprecipitate PER2 from extracts of cells co-transfected with *Per2 *and HA-tagged *Clock *(Fig. 2B). Our results hint at a binding of PER2, but not CRY1 or CRY2, to CLOCK. PER2-CLOCK interactions have been observed in the SCN and piriform cortex as well [23].

CRY, especially CRY1, binding to BMAL1 has been described many times in various systems. For CRY binding to CLOCK, however, contradictory findings have been published. Griffin *et al*. do not observe clear interactions between CRY1/2 and CLOCK in a yeast two-hybrid system [6], whereas Shearman *et al*. do [18]. Kiyohara *et al*. [15] report that they were not able to co-immunoprecipitate CLOCK with CRY1 in the absence of BMAL1, and also in other cases BMAL1/CLOCK(/PER2)/CRY1 complexes, rather than individual components, have been used to characterize interactions [17,24]. In the SCN and the piriform cortex, though, CRY1 co-immunoprecipitated with CLOCK [23], and the same group was able to demonstrate CRY1 binding to CLOCK in co-transfected HeLa cells. Therefore, it appears likely that in this case the respective results depend strongly on the system and cell type used and that cell-specific factors might be involved in mediating the interaction. In the mammalian two-hybrid system we employed in HER911 cells, CRY1 and 2 do not interact with CLOCK.

### CRY1 and CRY2 influence the interaction between PER2 and BMAL1

**Figure 3:**
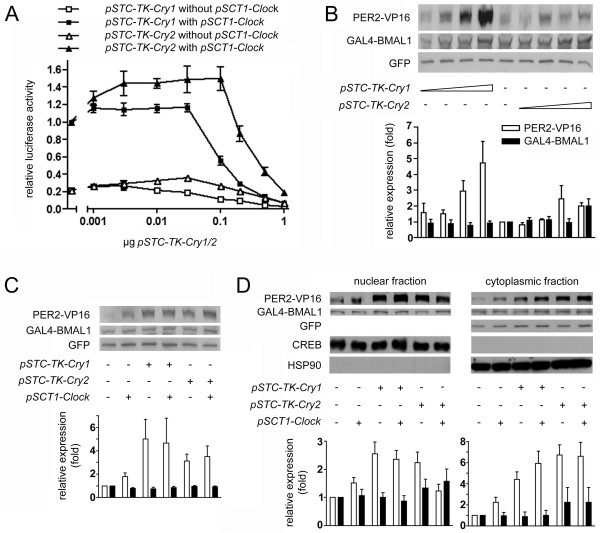
**CRY1 and CRY2 have different effects on PER2-BMAL1 interaction**. (A) HER911 cells were co-transfected with *pFR-luc*, *pCMV-lacZ*, *pSCT1-Per2-VP16*, *pSCT1-Gal4-Bmal1 *and the indicated doses of *pSTC-TK-Cry1/2 *with or without addition of 0.3 μg *pSCT1-Clock*. For each experiment (n = 3), values obtained for cells transfected with *pSCT1-Clock *but without *pSTC-TK-Cry *were set to 1. (B), (C) and (D) HER911 cells were co-transfected with *pSCT1-Per2-VP16*, *pSCT1-Gal4-Bmal1*, *pEGFP-N3 *and 0.001, 0.01, 0.1 or 1 μg *pSTC-TK-Cry1/2 *(B) or 0.1 μg *pSTC-TK-Cry1/2 *with and without addition of 0.3 μg *pSCT1-Clock *(C, D). The amount of PER2-VP16, GAL4-BMAL1 and GFP was determined by Western blotting of total lysates (B, C) or nuclear and cytoplasmic fractions (D). Antibodies against CREB and HSP90 were used to verify correct cell fractionation. PER2-VP16 and GAL4-BMAL1 were normalized to GFP to correct for transfection efficiency. For both proteins, values obtained for cells transfected with *pSCT1-Per2-VP16*, *pSCT1-Gal4-Bmal1 *and *pEGFP-N3 *only were set to 1 for each experiment (n = 4 for B and D, n = 5 for C). Blots are representative results from one experiment.

Since in our system, all three repressors bind to BMAL1, we wanted to know whether these proteins mutually influence each other's binding to BMAL1. We first analyzed the influence of CRY1 and 2 on the interaction between PER2 and BMAL1. HER911 cells were co-transfected with *Gal4-Bmal1*, *Per2-VP16 *and different amounts of *Cry1/2 *expression vectors (1 ng-1 μg). For *Cry1*, we observed only a very slight increase in reporter luciferase activity when low amounts (1–3 ng) of the plasmid were co-transfected, starting from 100 ng luciferase activity decreased markedly (Fig. 3A, open squares). When we co-transfected *Cry2*, however, the increase in reporter activity was markedly higher and sustained over a much wider dose range (1–100 ng), a decrease was observed only with the two highest amounts of co-transfected plasmid (Fig. 3A, open triangles).

Our results indicate that CRY1 and CRY2 have different effects on PER2-BMAL1 interaction. We wished to find out whether this would still be true in the presence of CLOCK and repeated the co-transfections as described above, but additionally co-transfected *Clock*. We observed an overall higher reporter activity but were able to reproduce the differences between *Cry1 *(Fig. 3A, solid squares) and *Cry2 *(Fig. 3A, solid triangles). A two-way ANOVA analysis of the data showed highly significant differences between *Cry1 *and *Cry2 *(p < 0.001), a highly significant dose-dependence (p < 0.001) and an equally highly significant interaction between the two parameters (p < 0.001).

To exclude that the modulations of reporter activity were only due to an increase or decrease in GAL4-BMAL1 or PER2-VP16 expression, we analyzed the total expression levels of the two proteins by Western blot. Co-transfection of 1 ng-1 μg *Cry1 *expression plasmid led to a dose-dependent increase in PER2-VP16 expression whereas it did not influence GAL4-BMAL1 levels, co-transfection of *Cry2 *caused a slight increase in both PER2-VP16 and GAL4-BMAL1 expression only with the highest amount tested (Fig. 3B). Additional co-transfection of *Clock *had no effect on GAL4-BMAL1 levels. PER2-VP16 expression levels were slightly elevated when *Clock*, but not *Cry*, was co-transfected whereas no further increase was seen with *Clock *in the presence of *Cry *(Fig. 3C).

A crucial role of CRY proteins in the nuclear entry of PER1/2 has been reported [7]. It has also been described that CRY expression has an effect on BMAL1 localization [14]. Since in the mammalian two-hybrid system used in this study only fusion proteins that are present in the nucleus can activate the transcription of the reporter, we determined the subcellular localization of GAL4-BMAL1 and PER2-VP16. To this end, we performed Western blots on nuclear and cytoplasmic fractions of HER911 cells transfected as described above (Fig. 3D). The effects of *Cry1/2 *and *Clock *basically followed the pattern observed in total extracts for both fractions, we did not detect any gross redistributions of the fusion proteins. If anything, the influence was stronger in the cytoplasm, which would not influence mammalian two-hybrid results.

In summary, fusion protein expression levels do not correlate with the degree of reporter activation, since e.g. the highest amount of *Cry1 *leads to a marked increase in PER2-VP16 expression and nonetheless significantly decreases luciferase activity. We therefore conclude that the modulations of luciferase activity we observe in the mammalian two-hybrid system reflect true modulations of PER2-BMAL1 interaction by the respective co-expressed proteins. In this scenario, CLOCK would strengthen the interaction because the increase in luciferase activity caused by co-transfection of *Clock *(Fig. 3A) is markedly higher than the slight increase in PER2-VP16 expression. A similar effect has in fact been observed by Kiyohara *et al*. for the interaction of CRY1 with the heterodimer [15]. Given that in our system, PER2 interacts with both BMAL1 and CLOCK (and those two in turn with each other), a stabilization of PER2-BMAL1 interactions by CLOCK might be envisaged through the formation of a heterotrimer where each component interacts with the other two. The increase in PER2-VP16 expression after co-transfection of *Cry1 *is very likely due to a stabilization of PER2-VP16 by CRY1, an effect that has been reported before [18,25,26].

CRY1 appears to be more effective than CRY2 in disrupting PER2-BMAL1 interactions. The observed decrease in luciferase activity is not due to less PER2-VP16 or GAL4-BMAL1 available, on the contrary, there is clearly elevated PER2-VP16 expression. For *Cry2*, a less pronounced increase in expression is observed, especially with low amounts of co-transfected *Cry2*, which, however, already cause a marked increase in reporter activation. Of course, these effects might be cell-specific; however, they fit in with previous observations. Recently, CRY1 has been proposed as the main repressor of BMAL1-CLOCK-mediated transcription [15,24], and previous studies in mice also show that the *Cry1 *gene has a dominant role over *Cry2*, because one normal *Cry1 *allele sustains normal circadian rhythms in behavior, while one *Cry2 *allele leads to arrhythmicity [20]. Since the PER proteins have been reported to be weaker repressors than CRY1/2 [7,27], the ability of CRY1 (and, to a lesser extent, CRY2) to disrupt PER2-BMAL1 interactions might be important to allow stronger repression of the transcription activation potential of the heterodimer.

### PER2 does not significantly affect the interaction between CRY and BMAL1

**Figure 4:**
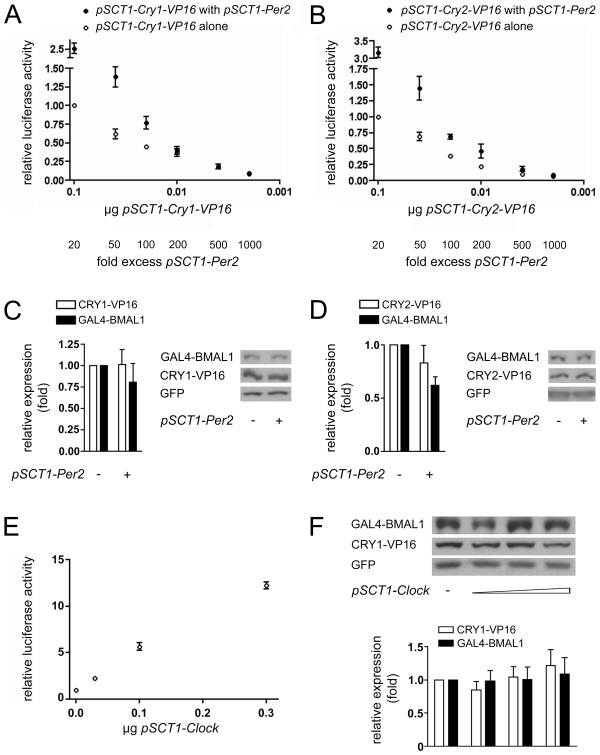
**Influence of PER2 and CLOCK on CRY1/2-BMAL1 interactions**. (A) and (B) HER911 cells were co-transfected with *pFR-luc*, *pCMV-lacZ*, *pSCT1-Gal4-Bmal1 *and 2 μg *pSCT1-Per2*. To obtain the indicated fold excess of *pSCT1-Per2*, 0.1, 0.04, 0.02, 0.01, 0.004 and 0.002 μg *pSCT1-Cry1-VP16 *(A) or *pSCT1-Cry2-VP16 *(B) were co-transfected (solid circles). Each dose of *pSCT1-Cry1/2-VP16 *was also transfected in the absence of *pSCT1-Per2 *(open circles) to assess the effect of PER2 on CRY1/2-BMAL1 interactions. For each experiment (n = 3), values obtained for cells transfected with the highest amount of *pSCT1-Cry1/2-VP16 *but without *pSCT1-Per2 *were set to 1. Note that the amounts *pSCT1-Cry1/2-VP16 *transfected are reversely plotted on the X axis, so that fold excess *pSCT1-Per2 *increases from left to right (see numbers below the graphs). (C), (D) and (F) HER911 cells were co-transfected with *pSCT1-Gal4-Bmal1*, *pEGFP-N3 *and either *pSCT1-Cry1-VP16 *(C and F) or *pSCT1-Cry2-VP16 *(D). For (C) and (D), transfections were performed with or without 2 μg *pSCT1-Per2*, for (F) 0.1, 0.3 or 1 μg *pSCT1-Clock *were co-transfected. The amount of CRY1/2-VP16, GAL4-BMAL1 and GFP was determined by Western blotting of total lysates. CRY1/2-VP16 and GAL4-BMAL1 were normalized to GFP to correct for transfection efficiency. For all proteins, values obtained for cells transfected with *pSCT1-Cry1/2-VP16*, *pSCT1-Gal4-Bmal1 *and *pEGFP-N3 *only were set to 1 for each experiment (n = 3). Blots are representative results from one experiment. (E) HER911 cells were co-transfected with *pFR-luc*, *pCMV-lacZ*, *pSCT1-Gal4-Bmal1*, *pSCT1-Cry1-VP16 *and the indicated amounts of *pSCT1-Clock*. For each experiment (n = 3), values obtained for cells transfected with *pSCT1-Gal4-Bmal1 *and *pSCT1-Cry1-VP16 *only were set to 1.

If the CRY proteins, especially CRY1, act as the main repressors, PER2 should not be able to destabilize the interactions between the CRY proteins and BMAL1. Preliminary experiments showed that co-transfection of small amounts of *Per2 *indeed did not reduce CRY1/2-BMAL1-mediated luciferase activation in the mammalian two-hybrid system (data not shown). To further test this hypothesis, we co-transfected *Cry1/2-VP16 *together with *Gal4-Bmal1 *and an excess of *Per2*. Since the DNA amount that can be transfected is limited, we decreased the amounts of co-transfected *Cry1/2-VP16 *rather than increased that of *Per2*. For *Cry1 *(Fig. 4A) as well as for *Cry2 *(Fig. 4B) we found a dose-dependent reduction in luciferase activity as co-transfected plasmid amount decreased, both in the presence (solid circles) and the absence (open circles) of PER2. Co-transfection of *Per2 *actually increased luciferase activity instead of diminishing it, which might hint at a stabilization of CRY-BMAL1 interactions by PER2. Alternatively, it could be due to increased protein expression or nuclear availability of the fusion proteins; we were, however, not able to determine expression levels of the fusion proteins in this experimental set-up. The currently transfected amounts of *Cry1/2-VP16 *were too low to yield any signal in a Western blot, and we were not able to increase them in a way that would allow a detection of the lowest amount without exceeding the transfection limit with the highest amount, which is 50-fold higher. We tried to at least assess the influence of co-transfection of *Per2 *on the expression levels of GAL4-BMAL1 and CRY1/2-VP16 using only one amount of each expression plasmid. PER2 co-expression did not affect CRY1/2-VP16 levels and had only a small effect on GAL4-BMAL1 expression, which was slightly decreased (Fig. 4C and 4D). Consequently, the increase in luciferase activity observed when *Per2 *is co-transfected very likely is not due to elevated fusion protein levels.

Although in our system, we could not detect any interaction between CRY1 and CLOCK, it has been described in other systems, indicating that CLOCK might have an impact on CRY1-BMAL1 interaction. Indeed, when we co-transfected increasing amounts of *Clock *expression plasmid together with *Gal4-Bmal1 *and *Cry1-VP16*, we observed a dose-dependent increase in luciferase activity (Fig. 4E) whereas GAL4-BMAL1 and CRY1-VP16 expression levels remained almost constant (Fig. 4F). CLOCK thus appears to stabilize the interaction, possibly by inducing conformational changes in BMAL1 that facilitate CRY1 binding. As for PER2, a heterotrimer might be formed that is more stable than the CRY1-BMAL1 complex alone, even in the absence of direct CRY1-CLOCK interactions. The results of Kiyohara *et al*. [15] support a heterotrimer formation as well, since they co-immunoprecipitate CRY1 with CLOCK only in the presence of BMAL1.

Lee *et al*. report impaired nuclear translocation of CRY proteins in the livers of *Per1*/*Per2 *double mutant mice [19], and it has been demonstrated that PER2 lacking the nuclear localization signal can retain CRY proteins in the cytoplasm [28]. However, overexpressed CRY1 and 2 have been shown to be nuclear proteins in cells [7]. Co-transfection of (full-length) *Per2 *should therefore in principle not influence the sub-cellular localization of at least the CRY proteins. To our knowledge, no dependence of sub-cellular localization of BMAL1 on PER2 has been reported, either, and since BMAL1 is a predominantly nuclear protein [19], its nuclear availability should not drastically vary. Thus, we did not perform any cell fractionations for CRY1/2-VP16 and GAL4-BMAL1.

### PER2, CRY1 and CRY2 bind different domains of BMAL1

To map the regions of the BMAL1 protein that are critical for interactions with CRY1/2 and PER2 we constructed three deletion mutants of *Bmal1 *fused to the Gal4 DBD (*Gal4-Bmal1ΔHLH/PAS A*; *Gal4-Bmal1ΔPAS B/C-term *and *Gal4-Bmal1ΔC-term*). We confirmed that their expression levels were comparable to those of full-length GAL4-BMAL1 (data not shown) and tested their capacity to activate the luciferase reporter in the mammalian two-hybrid system when co-expressed together with PER2-VP16, CRY1-VP16 or CRY2-VP16, respectively.

**Figure 5:**
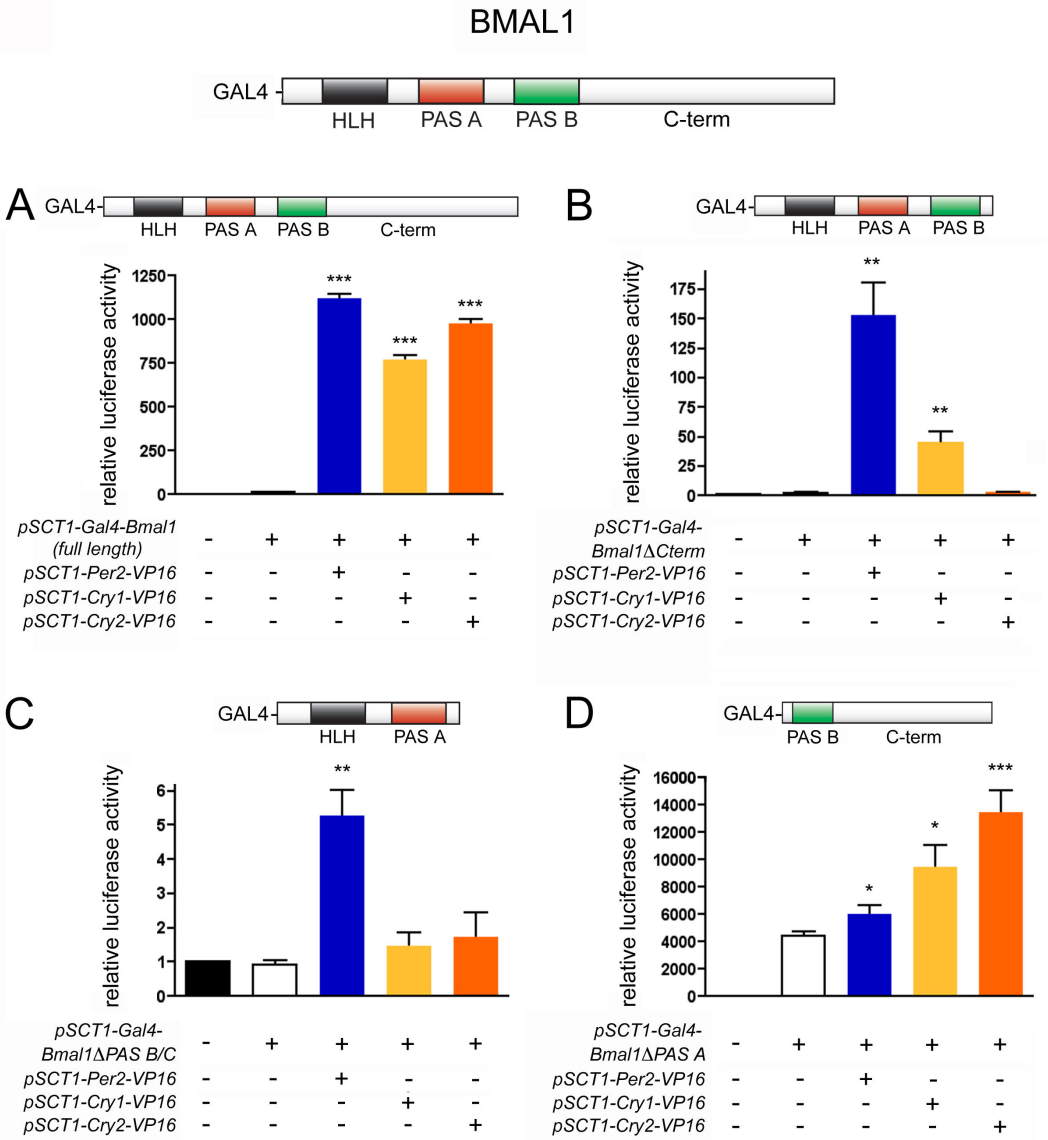
**Mapping of PER2, CRY1 and CRY2 binding sites in BMAL1**. HER911 cells were co-transfected with *pFR-luc *and μg *pCMV-lacZ *together with the indicated expresssion plasmids. For each experiment (n = 5–7), values obtained for cells transfected with the luciferase reporter alone were set to 1. Significant luciferase reporter activation by *pSCT1-Per2/Cry1/Cry2-VP16 *as compared to the respective *pSCT1-Gal4-Bmal1 *construct alone was determined by student's t-test (* p < 0.05, ** p < 0.01, *** p < 0.001).

Co-transfection of *Gal4-Bmal1ΔC-term *with *Per2-VP16 *led to a strong increase in luciferase activity (150-fold that of the reporter alone), indicating that the C-terminus of BMAL1 is not essential for PER2 binding. *Cry1-VP16 *was also able to augment luciferase activity significantly above background; however, the effect was not as strong as that observed with *Per2-VP16 *(50-fold). No significant increase was observed for *Cry2-VP16*. We therefore conclude that CRY1 is still able to weakly interact with this truncated version of BMAL1, whereas CRY2 binding is completely abolished (Fig. 5B).

When we tested *Gal4-Bmal1ΔPAS B/C-term*, a deletion mutant lacking not only the C-terminus but also the PAS B domain, only co-transfection with *Per2-VP16 *led to a slight (5-fold) elevation in luciferase activity. Neither *Cry1-VP16 *nor *Cry2-VP16 *had any statistically significant effect (Fig. 5C). PER2 consequently still appears to be able to bind to this BMAL1-mutant, although to a much lesser extent than to GAL4-BMAL1ΔC-term, which indicates that the PAS domain of BMAL1 might be involved in PER2 binding. It also seems to play a role in CRY1 binding since the residual reporter transactivation observed with GAL4-BMAL1ΔC-term and CRY1-VP16 disappears when the deletion in *Bmal1 *is extended to the PAS B domain.

GAL4-BMAL1ΔHLH/PAS A was able to activate the luciferase reporter to a considerable extent already in the absence of any VP16 fusion protein. Co-transfection with all three *VP16 *fusion constructs thus caused only slight, but nonetheless significant, increases in luciferase activity (1.5-fold for *Per2-VP16*, 2.5-fold for *Cry1-VP16 *and 3-fold for *Cry2-VP16 *as compared to *Gal4-Bmal1ΔHLH/PAS A *alone; Fig. 5D). Although it is difficult to draw any conclusions due to the high background activity of this *Bmal*1 mutant, these results confirm the observations made using the other two truncated forms, namely that the N-terminal portion of BMAL1 appears to be most important for PER2 binding, less crucial for the binding of CRY1 and not essential for interactions with CRY2.

In summary, our interaction data leads to a model where PER2 binds to the N-terminus of BMAL1, where the PAS A domain is located. Since PER2 itself also contains a PAS domain, a direct interaction between these two domains could be envisaged. CRY1 would bind more towards the PAS B domain and CRY2 even further C-terminally. This hypothesis is in line with observations of Kiyohara *et al*. [15] who report normal binding of PER2, but not of CRY1, to a C-terminally truncated version of BMAL1. Others have also identified mutations in the C-terminus of BMAL1 that weaken BMAL1-CRY1 interactions [24]. To our knowledge, the binding site for CRY2 has not been mapped in BMAL1 so far. However, given that CRY2 was less effective than CRY1 in disrupting PER2-BMAL1 interactions in our system, it can be expected to be further away than the binding site for CRY1, which, in our model, would actually be the case.

CBP and p300, transcriptional co-activators found to interact with BMAL1, have also been hypothesized to bind to the extreme C-terminus that harbors a putative transcription activation domain [13]. Thus, the fact that CRY1 and CRY2 bind more C-terminally to BMAL1 than PER2 might also explain why they have a stronger capacity to inhibit BMAL1-CLOCK-mediated transcriptional activation.

### Confirmation of the interactions in cos-7 cells

We were not able to confirm the interactions identified in the mammalian two-hybrid system by co-immunoprecipitation of *in vitro *transcribed/translated proteins (data not shown). This might be due either to the presence of bridging proteins in HER911 cells or to post-translational modifications of the interaction partners, which are vital for circadian clock function *in vivo *[19]. We tried to precipitate one *in vitro *expressed interaction partner together with one expressed in HER911 cells in order to reconstitute potential post-translational modifications on at least one of the proteins. However, in our hands, this was not possible either (data not shown), indicating that modifications of both partners might be necessary for interactions.

**Figure 6:**
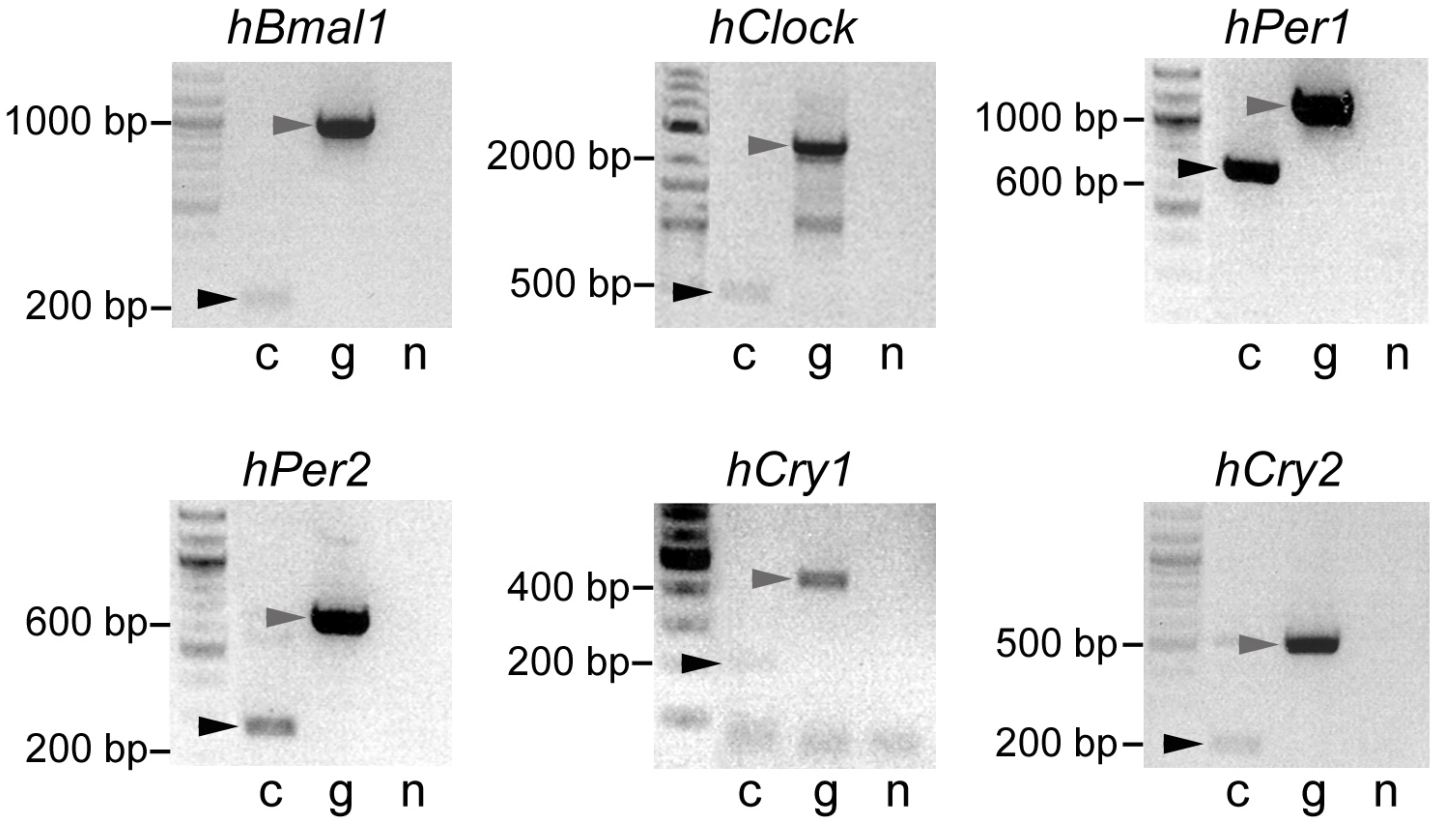
**HER911 cells express endogenous clock genes on the mRNA level**. cDNA of HER911 cells was obtained by reverse transcription of total RNA. *hPer1*, *hPer2*, *hCry1*, *hCry2*, *hBmal1 *and *hClock *cDNAs were amplified by PCR (lanes c). As positive control and to distinguish between true cDNA products (black arrowheads) and products from contaminating genomic DNA (gray arrowheads), a parallel reaction was run using human genomic DNA as template (lanes g). A negative control was done with water (lanes n).

As the primary interaction partners of clock components are other clock components, we wanted to find out whether clock genes, whose products might act as bridging proteins, were endogenously expressed in HER911 cells. We performed RT-PCR for *hPer1*, *hPer2*, *hBmal1*, *hClock*, *hCry1 *and *hCry2 *and were indeed able to detect transcripts of all six genes (Fig. 6). We did not detect any expression on the protein level, however (data not shown). Still, this does not necessarily mean that they are truly absent because their levels might just be below the detection limit. Consequently, it cannot be excluded that endogenous oscillator components are present and contribute to the interactions observed in HER911 cells. We therefore tried to reproduce our results in cos-7 cells that have been reported to express hardly any endogenous clock genes [29].

**Figure 7:**
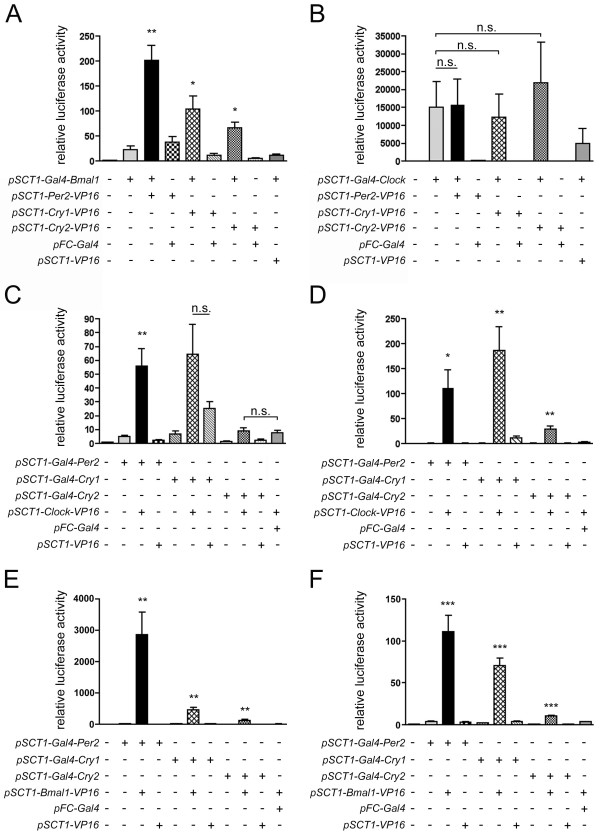
**Confirmation of the interactions in cos-7 cells**. cos-7 (A-C, E) and HER911 (D, F) cells were co-transfected with *pFR-luc*, *pCMV-lacZ *and the indicated expression plasmids. For each experiment (n = 3 for A and B, n = 6 for C-F), values obtained for cells transfected with the luciferase reporter alone were set to 1. * p < 0.05, ** p < 0.01, *** p < 0.001 as determined by student's t-test compared to the relevant controls (*Gal4 *fusion alone, *Gal4 *fusion and *pSCT1-VP16*, *VP16 *fusion and *pFC-Gal4*); n.s. not significant.

We performed the mammalian two-hybrid assay as described above to identify interactions of PER2-VP16, CRY1-VP16 and CRY2-VP16 with GAL4-BMAL1 and GAL4-CLOCK, respectively. As in HER911 cells, all three VP16 fusions were able to significantly increase luciferase activity when co-transfected with *Gal4-Bmal1 *(200-fold for *Per2-VP16*, 110-fold for *Cry1-VP16 *and 70-fold for *Cry2-VP16 *as compared to the reporter alone). This increment was not observed when they were co-expressed with the Gal4 DBD or when *Gal4-Bmal1 *was co-transfected with *VP16 *(Fig. 7A). Thus, PER2, CRY1 and CRY2 interact with BMAL1 also in cos-7 cells.

GAL4-CLOCK activated the reporter to such a high extent already in the absence of interaction partners that no further significant increase in luciferase activity could be detected upon co-expression of PER2-VP16, CRY1-VP16 or CRY2-VP16 (Fig. 7B).

To be able to analyze interactions with CLOCK, we constructed plasmids that encoded fusions of the GAL4 DBD to PER2, CRY1 and CRY2 and a fusion of VP16 to CLOCK and repeated the two-hybrid assay. Neither one of the GAL4 DBD fusion proteins caused a more than 10-fold increase in luciferase activity as compared to the reporter alone. When *Clock-VP16 *was co-transfected, significantly enhanced (55-fold) activity values were only observed for *Gal4-Per2*, indicating that indeed only PER2 interacts directly with CLOCK. Co-transfection of *Gal4-Cry1 *with *Clock-VP16 *also led to higher luciferase values; however, there were considerable inter-experimental variations, and moreover, luciferase activity was already elevated when *VP16 *alone was co-transfected. This hints at an interaction between CRY1 and VP16 rather than between CRY1 and CLOCK. The difference in luciferase activity between co-transfection of *Clock-VP16 *and *VP16 *was not statistically significant (Fig. 7C).

We wished to confirm the results obtained using the new plasmids in HER911 cells and performed the same two-hybrid assay as in cos-7 cells. In this cell line, however, all three GAL4 DBD fusion proteins in combination with CLOCK-VP16 significantly augmented reporter activity levels (110-fold for GAL4-PER2, 190-fold for GAL4-CRY1 and 30-fold for GAL4-CRY2 as compared to the reporter alone; Fig. 7D).

Since the interaction between CRY2 and CLOCK can only be observed in HER911 cells, and only when using GAL4-CRY2 and CLOCK-VP16 but not with GAL4-CLOCK and CRY2-VP16 (Fig. 2D), we think that it might actually be an artefact arising from an interaction of CRY2 with endogenous PER2 and/or BMAL1 that, in turn, interacts with CLOCK-VP16. Since for CRY1 and CLOCK, the results were not quite clear in cos-7 cells, either, we co-transfected HER911 cells with *Cry1 *and *HA-Clock *and tried to co-immunoprecipitate CRY1 with an anti-HA antibody, which did not work, however (data not shown). We therefore believe that also this alleged direct interaction is rather indirect and mediated by endogenous PER2 and/or BMAL1.

Co-transfection of the newly generated *Gal4 *fusion constructs with *Bmal1-VP16 *confirmed the results obtained previously both in HER911 and in cos-7 cells. All three proteins, GAL4-PER2, GAL4-CRY1 and GAL4-CRY2, caused a significant increased in luciferase activity when co-expressed together with BMAL1-VP16 (cos-7: 2900-fold for GAL4-PER2, 450-fold for GAL4-CRY1 and 125-fold for GAL4-CRY2, Fig. 7E; HER91: 110-fold for GAL4-PER2, 70-fold for GAL4-CRY1 and 10-fold for GAL4-CRY2, Fig. 7F).

In summary, our two-hybrid assays in cos-7 cells confirm the interactions identified using the same system in HER911 cells, namely PER2-BMAL1, PER2-CLOCK, CRY1-BMAL1 and CRY2-BMAL1. We still cannot exclude the involvement of endogenous bridging proteins that are present in both cell lines. However, the fact that the interactions still take place in a cell line devoid of endogenous clock genes strongly argues against the notion that endogenous clock components are necessary to stabilize the observed interactions.

## Conclusion

**Figure 8:**
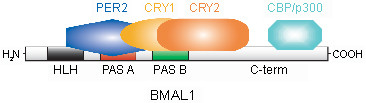
**Model of PER2, CRY1 and CRY2 binding to BMAL1**. Proposed binding sites for PER2, CRY1 and CRY2 in the BMAL1 protein based on the interaction data shown in Fig. 5. Note that CRY1 and 2 bind closer to the C-terminus, which has been described as a potential interaction site for the activators CBP/p300 (see text).

In the present study we identify interactions between BMAL1 and CLOCK, the two components of the positive feedback loop of the mammalian circadian clock, and the repressors PER2, CRY1 and CRY2. We show that PER2 binds to both BMAL1 and CLOCK whereas in our system, CRY1 and CRY2 are only able to bind to BMAL1. These interactions can be observed in transfected cells also in the absence of endogenous clock proteins, but not *in vitro*, indicating that post-translational modifications of the interaction partners and/or not clock-related bridging proteins are necessary to stabilize them. Analysis of deletion mutants of BMAL1 reveals that PER2 interacts with N-terminal regions, in contrast to CRY1 and CRY2 that both need C-terminal motifs to be able to bind to BMAL1 (Fig. 8). The CRY proteins moreover seem to have a higher affinity to BMAL1 than PER2. Taken together, our results provide new insights into the interactions between activating and repressing components of the circadian clock. They also confirm the notion that the CRY proteins are more potent inhibitors of BMAL1-CLOCK mediated transcriptional activation than the PER proteins, which might be due both to their higher affinity to BMAL1 and their binding to the C-terminus of BMAL1.

## Methods

### Plasmids

Full-length mouse cDNAs encoding BMAL1 [EMBL:BC011080], CLOCK [EMBL:AF000998], and PER2 [EMBL:AF036893] were cloned into *pSCT1*, a *pUC18*-based expression vector carrying the CMV promoter and intron 2, exon 3, and the 3' UTR of the *β-globin *gene to enhance expression [30]. The fusion constructs for BMAL1-GFP, GAL4-BMAL1, GAL4-CLOCK, GAL4-PER2, GAL4-CRY1, GAL4-CRY2, CLOCK-VP16, PER2-VP16, CRY1-VP16, CRY2-VP16, PAX5-VP15 and the VP16 tag alone were expressed from the same vector. *pSCT1 *was also used to express full-length bacterial β-galactosidase and chloramphenicol acetyl transferase [31]; these constructs will be referred to as *pCMV-lacZ *and *pCMV-CAT*, respectively.

To construct the *Bmal1 *deletions fused to *Gal4*, a 1051 bp *Hin*dIII-*Bam*HI fragment was excised from *pSCT1-Gal4-Bmal1 *to yield *pSCT1-Gal4-Bmal1ΔPAS BΔC-term *(aa 1–278 of the full-length protein). Excision of a 1912 bp *Bsr*FI-*Hin*dIII fragment from the same vector gave rise to *pSCT1-Gal4-Bma1lΔHLHΔPAS A *(aa 276–625), and a 488 bp *Sph*I-*Bam*HI fragment was excised to construct *pSCT1-Gal4-Bmal1ΔC-term *(aa 1–467).

For the expression of CRY1 [EMBL:AF156986] and CRY2 [EMBL:AF156987], the respective full-length mouse cDNAs were cloned into *pSTC-TK*, an expression vector similar to *pSCT1*, which additionally contains a thymidine kinase leader sequence after the CMV promoter. This vector was also used to express HA-CLOCK.

GFP was expressed from *pEGFP-N3 *(Clontech, Saint-Germain-en-Laye, France). The Gal4 DNA binding domain was expressed from a modified version of *pFA-CMV *(Stratagene, Amsterdam, The Netherlands), which will be referred to as *pFC-Gal4*. *pFR-luc *(Stratagene, Amsterdam, The Netherlands) is a GAL4-based luciferase reporter vector containing the luciferase gene under the control of a promoter with 5 GAL4 binding sites.

### Cell culture and maintenance

HER911 human retinoblastoma cells [32] and cos-7 African green monkey kidney cells [33] were routinely cultured in DMEM High Glucose supplemented with 10% FCS, 2 mM L-glutamine, and 100 U/ml penicillin/streptomycin (all from Bioconcept, Allschwil, Switzerland) at 37°C in a humidified atmosphere containing 5% CO_2 _and split when confluent.

### Reporter gene assays

Cells were seeded in 5 cm dishes, grown to 70% confluency and transfected with the calcium phosphate co-precipitation method. All transfection mixtures excluding those for mock transfections contained 3 μg *pFR-luc*, 0.1 μg *pCMV-lacZ *to allow normalization of the luciferase activity values. Unless indicated otherwise, 0.1 μg were transfected for *pSCT1-Gal4-Bmal1 *and its deletion mutants, *pSCT1-Cry1/2-VP16, pSCT1-Gal4-Per2, pSCT1-Clock-VP16*, *pSCT1-Bmal1-VP16*, 0.3 μg for *pSCT1-Gal4-Clock *and 0.5 μg for *pSCT1-Per2-VP16*. For *pFC-Gal4*, *pSCT1-VP16 *and *pSCT1-Pax5-VP16*, an amount corresponding to that of the respective *Gal4 *or *VP16 *fusion construct tested was used.*pCMV-CAT *was used to obtain the same amount of CMV promoter/enhancer in all samples. The total DNA amount was brought to 10 μg with calf thymus DNA. The volume was brought to 125 μl, 125 μl 0.5 M CaCl_2 _in 0.1 M Hepes, pH 7.0 were added, and samples were mixed well. After addition of 250 μl 0.75 mM Na_2_HPO_4_/0.75 mM NaH_2_PO_4_/.28 M NaCl in 0.05 M Hepes, pH 7.0, samples were mixed again and incubated for 1 minute at room temperature before adding the transfection mixture to the cells. Cells were incubated overnight, washed twice with TBS, supplied with fresh medium, incubated for another 24 h and subsequently lysed in 10% glycerol/10 mM MgAc/0.2% Triton X-100 in 50 mM Tris-HCl, pH 8.0.

Luciferase activity was measured according to [34]. 10 μl lysate were added to 100 μl 1 mM ATP (Sigma, Buchs, Switzerland)/10 mM MgAc/0.1 mg/ml BSA in 250 mM Tris-HCl, pH 7.5. Samples were injected with 100 μl 200 μg coenzyme A/30 μg luciferine (both Sigma, Buchs, Switzerland)/ml in 12.5 mM PIPES, pH 6.5 and light emission was measured after a delay of 0.3 seconds during a 10 second interval in a MicroLumatPlus luminometer (Berthold Technologies, Bad Wildbach, Germany).

β-galactosidase activity was measured as described in [35]. 10 μl lysate diluted 1:10 in lysis buffer were incubated with 250 μl 1 mg/ml MUG (Sigma, Buchs, Switzerland)/DMF in 90 mM Na_3_PO_4_/18 mM MgCl_2_, pH 8.0 for 20 minutes at 37°C protected from light. The reaction was stopped by addition of 100 μl 120 mM glycine/6 mM EDTA, pH 11.5, and the fluorescence of the samples was measured at 360 nm excitation and 460 nm emission wave length in a Lambda Fluoro 320 fluorimeter (MWG Biotech, Ebersberg, Germany).

For statistical analysis, values obtained for mock-transfected cells were subtracted from all other values. Subsequently, luciferase activity was normalized to β-galactosidase activity to correct for transfection efficiency. All experiments were performed at least three times, samples were measured in duplicates.

### Preparation of total, nuclear and cytoplasmic extracts

Cells were seeded in 5 cm dishes for total extracts or in 10 cm dishes for IPs and fractionations, grown to 70% confluency and transfected with linear polyethylenimine of 25 kDa (LINPEI25, Polysciences Europe, Eppelheim, Germany). Again, *pCMV-CAT *was used to obtain equal amounts of CMV promoter in each sample. For IPs, 10 μg *pSCT1-Bmal1-GFP *and *pSCT1-HA-Clock *and 5 μg *pSCT1-Per2*, *pSTC-TK-Cry1 *and *pSTC-TK-Cry2 *were transfected. For fractionations and total extracts that were not used for IPs, 1 μg *pSCT1-Per2-VP16*, 3 μg *pSCT1-Cry1/2-VP16 *and 0.5 μg *pSCT1-Gal4-Bmal1 *were used for 5 cm dishes; for 10 cm dishes, amounts were increased 3-fold. 0.2 μg *pEGFP-N3 *were included in each transfection to control transfection efficiency and to allow normalization of expression levels. Plasmid DNA was brought to 200 μl with 150 mM NaCl in 20 mM HEPES pH 7.4 and mixed with 13 equivalents of LINPEI25. Samples were incubated for 10 minutes at room temperature before adding the DNA-LINPEI25 complexes directly to the culture medium. After 6 hours cells were washed once with TBS, supplied with fresh medium and incubated for another 24 hours before lysis.

For total extracts, cells were lysed in 1 mM EDTA/150 mM NaCl/1% Triton X-100/10% glycerol/0.05% β-mercaptoethanol/protease inhibitor cocktail (complete EDTA-free; Roche, Rotkreuz, Switzerland) in 20 mM Tris-HCl, pH 7.5.

Nuclear and cytoplasmic fractions were obtained according to [36]. Cells were incubated in 10 mM KCl/1.5 mM MgCl_2_/0.5 mM DTT/protease inhibitor cocktail in 10 mM HEPES-KOH, pH 7.9 for 15 minutes on ice. After centrifugation for 5 minutes at 1200 g and 4°C, the supernatant was stored as the cytosolic fraction. The pellet was washed twice with the same buffer and resuspended in 25% glycerol/0.42 M NaCl/1.5 mM MgCl_2_/0.2 mM EDTA/0.5 mM DTT/protease inhibitor cocktail in 20 mM Hepes-KOH, pH 7.9. Samples were incubated in rotation for 20 minutes at 4°C and centrifuged for 15 minutes at 13000 g and 4°C. The supernatant was stored as the nuclear fraction.

Protein concentration of total extracts or fractions was determined using the BioRad Protein Assay (BioRad, Reinach, Switzerland) according to manufacturer's instructions. 4× loading dye (4% SDS/4% β-mercaptoethanol/40% glycerol in 200 mM Tris-HCl, pH 6.8) was added to all samples and they were boiled before subjection to SDS-PAGE.

### Co-immunoprecipitation

Cells were transfected with linear polyethylenimine of 25 kDa as described above. For co-immunoprecipitations with BMAL1-GFP, 600 μg total protein were brought to a final volume of 800 μl with lysis buffer and incubated with 2 μg Anti-GFP antibody (Roche, Rotkreuz, Switzerland) and 50 μl protein G agarose beads (Roche, Rotkreuz, Switzerland) in rotation over night at 4°C. Beads were washed twice with lysis buffer and once with 250 mM NaCl/10% glycerol/0.1% NP-40 in 50 mM Tris-HCl, pH 7.5.

For co-immunoprecipitations with HA-CLOCK, 600 μg total protein were incubated with 0.5 μg anti-HA high affinity antibody (Roche, Rotkreuz, Switzerland) in rotation over night at 4°C. 50 μl protein G agarose beads were added and samples were incubated for another 3 hours at 4°C. Beads were washed three times using 0.1 mM EDTA/10% glycerol/1% Triton-X 100/0.3% β-mercaptoethanol in 30 mM Tris-HCl, pH 7.5 supplemented with 1 M, 0.1 M and no NaCl, respectively. In both cases, beads were resuspended in 2% SDS/2% β-mercaptoethanol/20% glycerol in 100 mM Tris-HCl, pH 6.8. Samples were boiled and subsequently subjected to SDS-PAGE.

### Western blot

Total lysates, cellular fractions or immunoprecipitates were separated by SDS-PAGE and transferred onto nitrocellulose membranes. Membranes were blocked for 1 h at room temperature in 5% milk/0.1% Tween-20 in TBS for PER2, CRY1, CRY2, GFP and BMAL1 and in 1% milk/0.1% Tween-20 in TBS for HA and incubated with primary antibodies diluted in blocking buffer at 4°C over night. Antibodies and dilution were anti-PER2 1:1000 (BD Biosciences, Allschwil, Switzerland for total lysates and immunoprecipitations; gift from J. Ripperger and S. Brown for fractions [8]), anti-BMAL1 1:1000 (gift from J. Ripperger and S. Brown [8]), anti-CRY1 1:500, anti-CRY2 1:750 (both Alpha Diagnostics, San Antonio, USA), anti-GFP 1:3000, anti-HA 1:1000 (both Roche, Rotkreuz, Switzerland). Membranes were washed and incubated with appropriate HRP-conjugated secondary antibodies (anti-rabbit, anti-mouse and anti-rat; all Sigma, Buchs, Switzerland) for 1 h at room temperature. Detection was performed using the Western blotting detection reagents kit (Amersham Biosciences, Freiburg, Germany) according to manufacturer's instructions. Membranes were exposed on Hyperfilm (Amersham Biosciences, Freiburg, Germany).

### RT-PCR

HER911 cells were seeded in 10 cm dishes and grown to 80% confluency. Cells were washed once with TBS and lysed in 1 ml RNA bee (AMS Biotechnology, Abingdon, UK) directly in the dish. Total RNA was isolated according to manufacturer's instructions and RNA integrity was checked on an agarose gel. cDNA was synthesized from 2 μg total RNA using SuperScript II (Invitrogen, Basel, Switzerland) according to manufacturer's instructions. Human genomic DNA (for primer optimization and as positive control) was from Promega (Wallisellen, Switzerland).

Primers for the amplification of human clock genes were 5'-CCCACCCCACCAGCCACTAC-3' and 5'-CCTGTGCCGGAGCGCGAGTC-3' for *hPer1 *(GenBank GeneID:5187), 5'-TGGATGTGGCTGTCTTGTAG-3' and 5'-GCCGGTGGATCTGCTCTGTG-3' for *hPer2 *(GenBank GeneID:8864), 5'-TGGATGTGGCTGTCTTGTAG-3' and 5'-TTTGGCTACCTTTTGGATAC-3' for *hCry1 *(GenBank GeneID:1407), 5'-AAGCGTTCCCCTCTCGATAC-3' and 5'-AGGGACAGATGCCAGTAGAC-3' for *hCry2 *(GenBank GeneID:1408), 5'-CATTCCTTCCAGTGGCCTAC-3' and 5'-GTCAACAGGGCCACCCAGTC-3' for *hBmal1 *(*hARNTL*; GenBank GeneID:406) and 5'-TCATCGGCAACAAGAAGAAC-3' and 5'-GCTTCCGGCTGCAGGCTGAG-3' for *hClock *(GenBank GeneID:9575). All primers were designed based on the genomic sequence so that amplicons would contain an intron to distinguish between products obtained from genomic and cDNA (1003/562 bp for *hPer1*, 590/237 bp for *hPer2*, 412/188 bp for *hCry1*, 511/202 bp for *hCry2*, 1017/213 bp for *hBmal1*, 2187/399 bp for *hClock*). 40 PCR cycles (30 seconds denaturation at 95°C, 30 seconds annealing, 2 minutes elongation at 72°C) were run, annealing temperatures were 62°C for *hPer1*, 60°C for *hPer2*, *hBmal1 *and *hClock*, 56°C for *hCry2 *and 50°C for *hCry1*.

## Authors' contributions

SL participated in the cloning of the expression plasmids used in this study and in designing and carrying out the two-hybrid assays, performed all Westerns and the statistical analysis and participated in drafting the manuscript. AB participated in the cloning and the design and performance of the two-hybrid assays. TT and SR provided all control and basic expression plasmids and cell lines and participated in the design of the cloning strategies and the two-hybrid assays. UA participated in the overall design of the study and in drafting the manuscript. All authors read and approved the final manuscript.
